# Sympathetic ophthalmia induced by vitrectomy for endogenous fungal endophthalmitis: a case report and literature review

**DOI:** 10.3389/fmed.2026.1863685

**Published:** 2026-07-07

**Authors:** Xiao-Yan Zhang, Jie Huang, Yan-E. Gao, Jiao Li, Ying Wen

**Affiliations:** 1 Ophthalmic Hospital Affiliated to Shandong University of Traditional Chinese Medicine, Jinan, China; 2 Shandong Provincial Key Laboratory of Integrated Traditional Chinese and Western Medicine for Prevention and Therapy of Ocular Diseases, Shandong Academy of Eye Disease Prevention and Therapy, Jinan, China

**Keywords:** case report, endophthalmitis, fungal endophthalmitis, sympathetic ophthalmia, vitrectomy

## Abstract

**Introduction:**

Sympathetic ophthalmia (SO) is a rare but serious inflammatory ocular disorder. We report a case of endogenous fungal endophthalmitis caused by *Aspergillus flavus* infection, which resulted in SO in the contralateral eye after two vitrectomy procedures.

**Case report:**

A 22-year-old man presented to our hospital with a 2-week history of redness and blurred vision in his right eye. Three months earlier, he had undergone two vitrectomy procedures for fungal endophthalmitis in his left eye, with culture results positive for *Aspergillus flavus*. Upon admission, antifungal therapy was administered; however, his health condition did not improve and progressively deteriorated. Metagenomic sequencing and microbial culture of intraocular fluid from the right eye revealed no fungi. Multimodal imaging, including optical coherence tomography (OCT), ocular B-scan ultrasonography, fundus examination, and indocyanine green angiography (ICGA), supported a definitive diagnosis of sympathetic ophthalmia. Treatment with prednisone and adalimumab stabilized the patient’s condition. During the 13-month follow-up period, the patient’s best-corrected visual acuity (BCVA) was 1.0 in the right eye and 0.04 in the left eye, with no observed recurrences.

**Conclusion:**

Sympathetic ophthalmia is a complex ocular disorder characterized by diverse clinical and imaging features, making early diagnosis and treatment challenging. This case underscores the importance of timely intervention and aggressive therapeutic strategies for managing this condition.

## 1 Introduction

Sympathetic ophthalmia (SO) is a bilateral granulomatous panuveitis that occurs following ocular trauma or intraocular surgery (1). The eye that experiences the initial injury is referred to as the “inciting” eye, while the other, unaffected eye is known as the “sympathizing” eye (2). Although SO is a rare condition, it poses a significant risk to vision. Its pathogenesis remains incompletely understood. The prevailing theory proposes that SO results from the development of a cell-mediated immune response against ocular antigens that are exposed during traumatic or surgical events (3, 4). Clinically, patients typically present with decreased best-corrected visual acuity (BCVA), ocular pain, redness, floaters, photophobia, photopsia, or metamorphopsia (5, 6). Traditionally, it was believed that a purulent ocular infection would destroy uveal tissue and its antigens to such an extent that the infected eye would be incapable of inciting SO (7). However, emerging evidence suggests that endophthalmitis fails to prevent the onset of SO and may actually potentiate its development (8). Nevertheless, there are few reports of SO caused by endogenous fungal endophthalmitis. Herein, we describe a case of SO that developed after vitrectomy for treating endogenous fungal endophthalmitis.

## 2 Case report

A 22-year-old man presented with a 2-week history of redness and blurred vision in the right eye. Three months earlier, following a 2-day fever, the patient had developed fungal endophthalmitis in the left eye. The patient underwent two 25-gauge complete vitrectomy surgeries within 1 week. In the first vitrectomy, balanced salt solution (BSS) was used as the tamponade agent. Because the infection persisted, a second vitrectomy was performed 5 days later, during which subretinal purulent material was removed, and silicone oil tamponade was placed. During this period, two intravitreal injections of voriconazole (1 mg/0.1 mL) were administered: one during the initial vitrectomy and another during the second vitrectomy, with a 5-day interval between them. The scleral incisions were sutured at each surgery. A*spergillus flavus* was isolated from the vitreous fluid using microbial culture. Oral voriconazole (200 mg twice daily) was continued for 3 months, after which the left eye infection was under control and the visual acuity was 0.04. His past medical history was notable only for fungal otitis media, which was the sole systemic illness.

**Figure 1:**
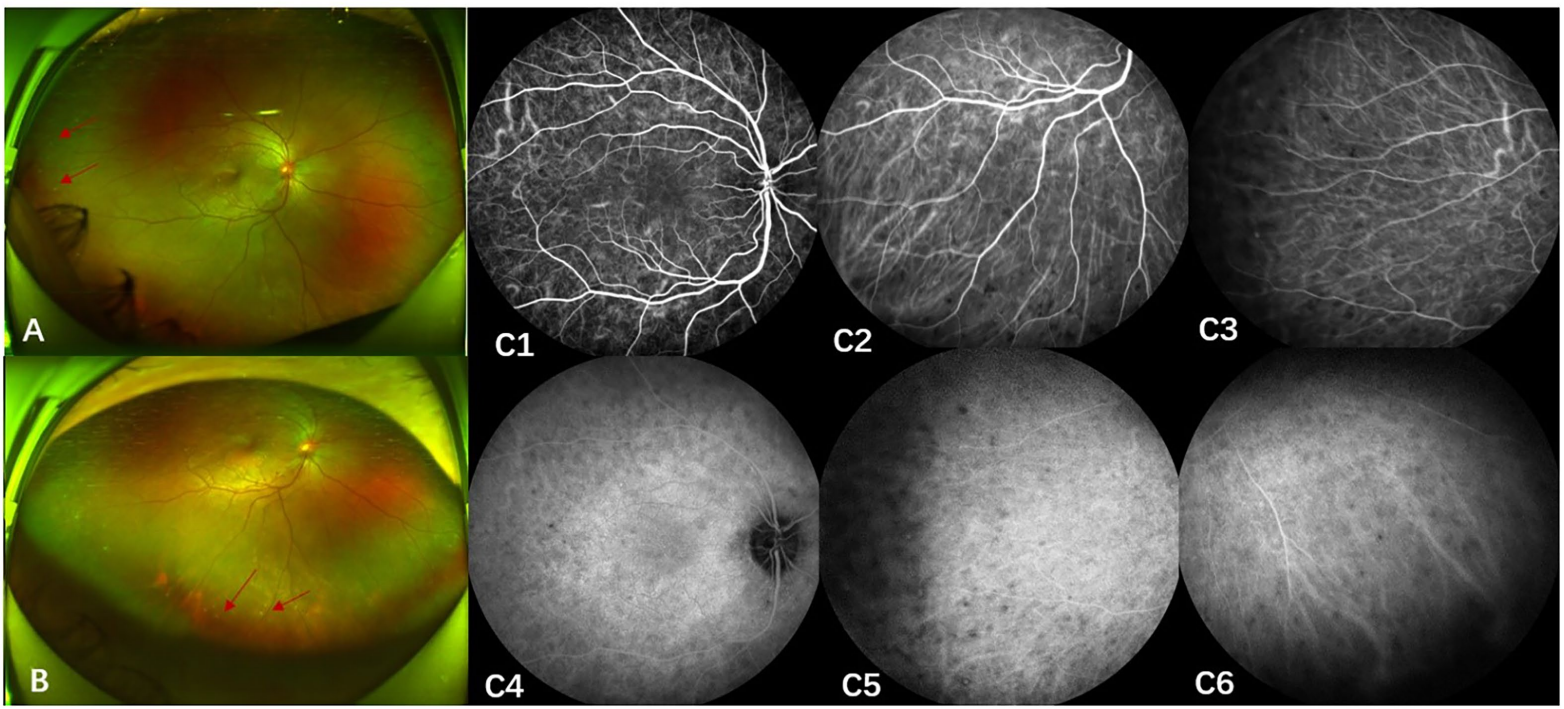
**(A,B)** Fundus image reveals radial folds in the macular area of the right eye and peripheral yellowish-white infiltrates. **(C1–C3)** ICGA reveals scattered punctate hypo-fluorescence in the early phase. **(C4–C6)** ICGA reveals scattered punctate hypo-fluorescence in the late phase.

**Figure 2:**
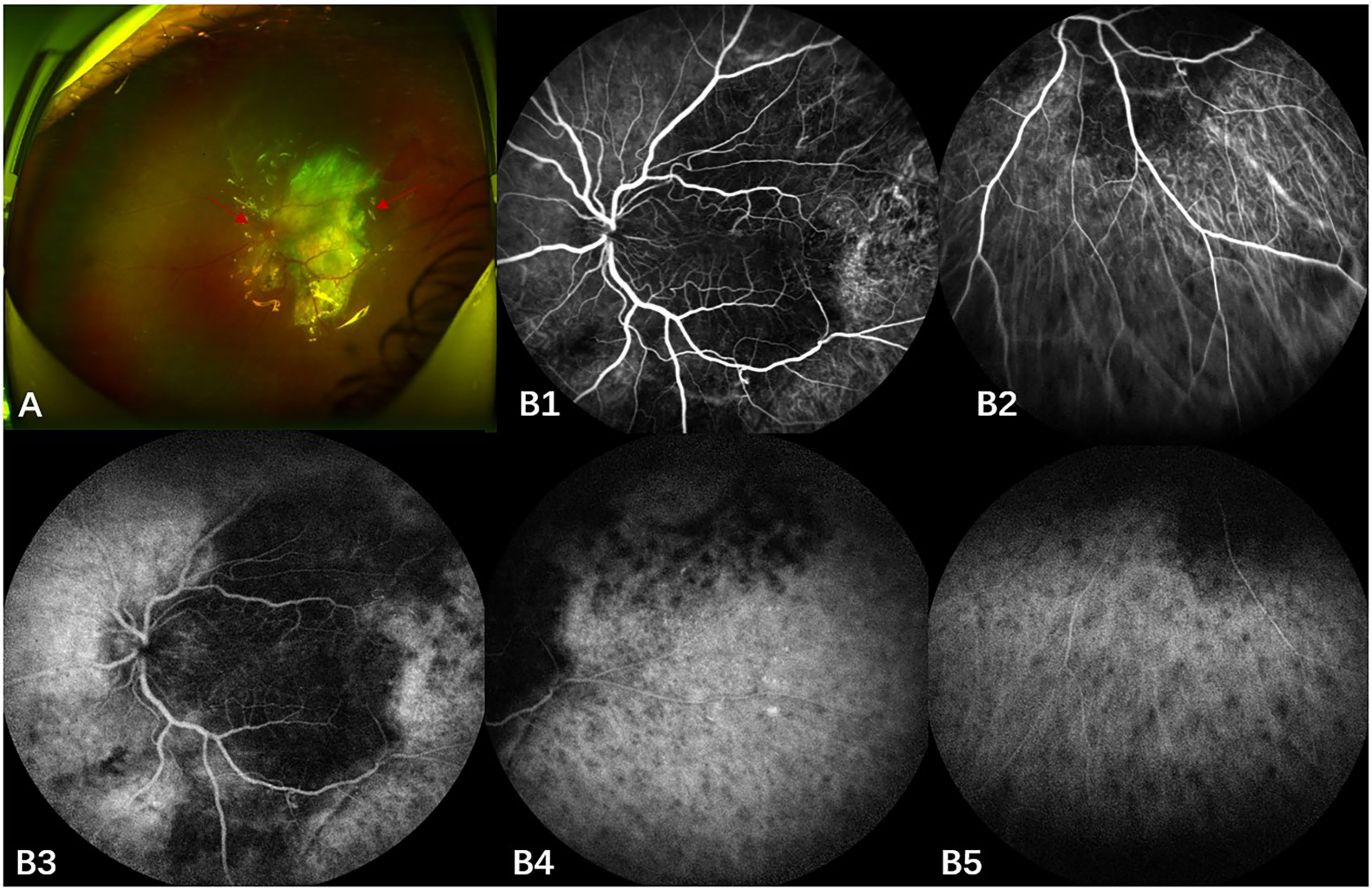
**(A)** Fundus image reveals optic disk hyperemia with indistinct margins, a yellowish-white macular scar in the left eye. **(B1,B2)** ICGA reveals a scar in the posterior pole, causing the blocking of fluorescence, along with scattered punctate hypofluorescence in the early phase. **(B3–B5)** ICGA reveals scattered punctate hypofluorescence in the late phase.

**Figure 3:**
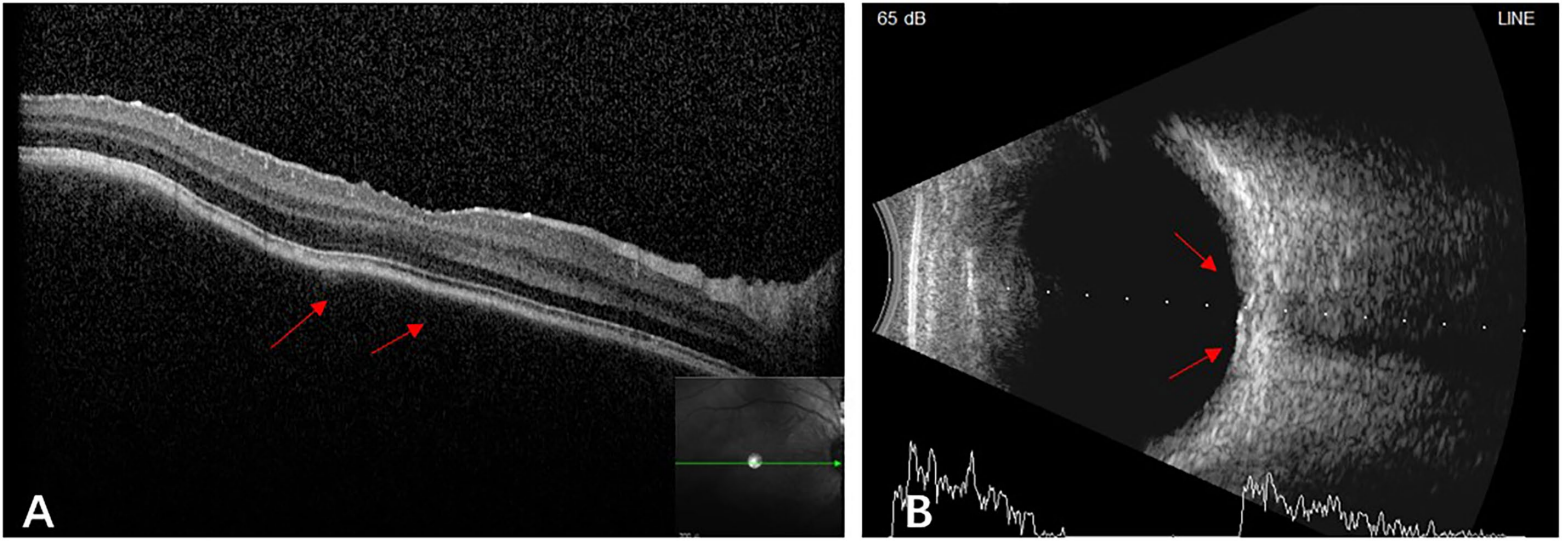
**(A)** OCT reveals ill-defined choroidal vascular structures with thickening. **(B)** B-scan shows retinal and choroidal edema in the right eye.

At presentation, BCVA was 0.8 in the right eye and 0.02 in the left eye. Intraocular pressure (IOP) was normal in both eyes. A slit-lamp and fundus examination of the right eye revealed conjunctival hyperemia, anterior chamber flare (+1), optic disk hyperemia with indistinct margins, radial folds in the macula, and widespread faint whitish deep retinal dots in the peripheral retina (Figures 1A,B). The left eye showed conjunctival hyperemia, anterior chamber flare (+1), silicone oil tamponade in the vitreous cavity, optic disk hyperemia with indistinct margins, a yellowish-white macular scar, and attached retina (Figure 2A). Fluorescence fundus angiography (FFA) was not performed since the patient was allergic to fluorescein sodium. Indocyanine green angiography (ICGA) showed punctate hypofluorescence in the choroid of the right eye during both the early and late phases (Figures 1C1–C6). In the left eye, a scar in the posterior pole blocked fluorescence, and scattered hypo fluorescence was observed in the peripheral retina during both the early and late phases (Figures 2B1–B5). Optical coherence tomography (OCT) indicated choroidal thickening in the macular area with indistinct vascular structures in the right eye (Figure 3A). B-scan ultrasonography showed retina and choroidal edema of the right eye (Figure 3B).

Given the patient’s history of fungal endophthalmitis, a fungal recurrence could not be completely ruled out. Therefore, while performing further intraocular fluid and systemic examinations to determine a definitive diagnosis, we administered voriconazole 200 mg intravenously twice daily. Aqueous humor samples were collected for metagenomic next-generation sequencing (m-NGS) and microbial culture to identify the causative pathogen, and a systemic evaluation was undertaken to determine the source of endophthalmitis. Hematologic tests revealed a mild leukocytosis, with no other abnormalities. Serological tests for syphilis were negative. The IDR tuberculin test was negative, as were immunological findings for anti-Borrelia IgM and IgG antibodies, p-ANCA, c-ANCA, anti-HBs antibody, HBs antigen, anti-HCV antibody, and HIV. Chest and abdominal computed tomography (CT) scans showed no abnormalities.

**Figure 4:**
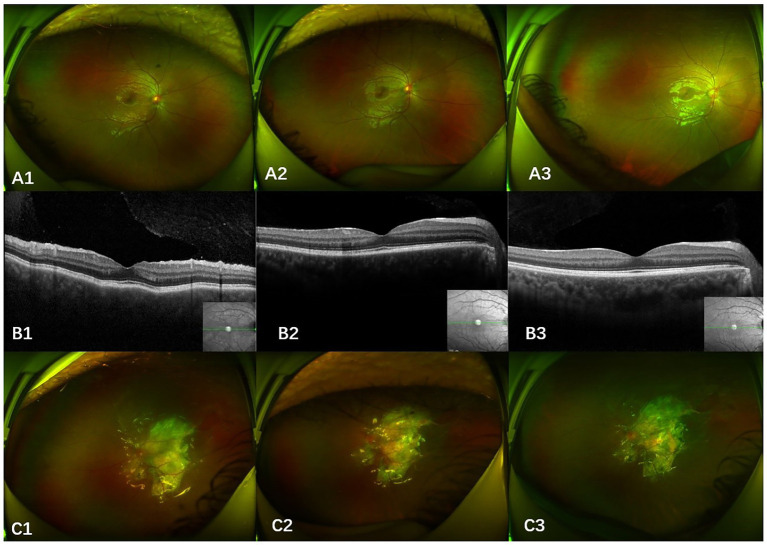
**(A1–A3)** Macular folds and yellowish-white spots in the right eye gradually resolved at 1 week, 1 month, and 3 months following treatment. **(B1–B3)** OCT at 1 week, 1 month, and 3 months after treatment showed decreased choroidal thickness and clearer vascular structures. **(C1–C3)** Optic disk and retinal edema in the left eye resolved at 1 week, 1 month, and 3 months after treatment.

After 3 days of antifungal therapy, the patient’s clinical condition deteriorated. The fundoscopic examination showed progressive optic disk swelling in both eyes. OCT revealed an increase in choroidal thickness in the macular area. The m-NGS of the aqueous humor showed negative results. Based on these imaging characteristics, negative microbiological findings, the absence of positive autoimmune serological markers, and the patient’s history of prior ocular surgery, fungal recurrence, and other causes of uveitis were reasonably excluded. Therefore, SO was diagnosed. After discontinuing intravenous voriconazole, the patient was given intravenous methylprednisolone 500 mg daily for 5 days. At discharge, he was transitioned to 90 mg of oral prednisone once daily. The oral prednisone was reduced using the following schedule: 90 mg daily for the first 2 weeks, followed by a 5 mg reduction every 2 weeks until a daily dose of 10 mg was reached; this dose was maintained for 1 month, then reduced to 5 mg daily and continued for one additional month before discontinuation. Adalimumab was injected subcutaneously every 2 weeks for 3 months. Eye drops were administered four times daily, with the frequency decreasing by one drop per day each week after the resolution of anterior chamber flare. One week after initiating this regimen, the patient’s blurred vision improved, and choroidal thickness was significantly reduced (Figure 4A1).

Anatomical changes were observed during follow-up, particularly on OCT and B-scan imaging. The white punctate infiltrates in the right eye gradually resolved at 1 week, 1 month, and 3 months (Figures 4A1–A3). OCT showed a progressive reduction in macular choroidal thickness in the right eye (Figures 4B1–B3). Optic disk edema in the left eye was resolved (Figures 4C1–C3). After 6 months of treatment, the macular structure in the right eye had completely normalized, and silicone oil was extracted from the left eye. BCVA was 1.0 in the right eye and 0.04 in the left eye. At 1-year follow-up, fundus examination revealed no apparent sunset glow fundus in either eye. No recurrence of ocular inflammation or treatment-related side effects were observed.

## 3 Discussion

The highest risk inciting event for SO is open-globe injury (9). In addition, a variety of ocular surgical procedures—including pars plana vitrectomy, trabeculectomy, cataract surgery, proton beam radiotherapy, corneal infections, and endophthalmitis—have been associated with the development of SO (10, 11). Here, we present a case with fungal endophthalmitis that progressed to SO following two vitrectomy procedures. The patient had no history of immunosuppression, surgery, or trauma; the only medical history relevant to endophthalmitis was fungal otitis media. Following the onset of fever, the fungus most likely spread hematogenously to the eye, resulting in endogenous infection. This case is atypical, and the triggering factors are likely multifactorial. Cumulative ocular trauma from two complete vitrectomies, two intravitreal injections, and silicone tamponade—all within 1 week—likely disrupted the blood–ocular barrier repeatedly, potentially serving as a trigger for SO in this patient. Following corticosteroid and immunomodulatory therapy, the inflammation showed rapid, sustained improvement and stabilization. This case serves as a clinical reminder that fungal endophthalmitis requiring multiple vitrectomy procedures within a short period may predispose patients to the subsequent development of SO, and clinicians should remain vigilant for this potential complication.

SO is defined as bilateral diffuse granulomatous uveitis (12). The inflammatory process typically begins in the choroid or anterior segment and progresses to panuveitis (13). The diagnosis is primarily clinical, based on symptoms including blurred vision, ocular pain, epiphora, photophobia, and redness (3, 14). Characteristic signs include granulomatous iridocyclitis with mutton-fat keratic precipitates, as well as variable anterior chamber cells and flare (15, 16). Posterior segment involvement may manifest as retinal vasculitis, choroiditis, or papillitis, with varying degrees of severity (17, 18). Multimodal imaging plays an essential role in supporting the diagnosis of SO and assessing inflammatory activity. ICGA is particularly valuable for detecting choroidal inflammation. The typical ICGA finding in SO is persistent, multifocal hypofluorescent spots from the early to late phases (19). These hypofluorescent areas correspond to sites of choroidal inflammatory cell infiltration and delayed filling, which are characteristic of SO. This finding not only supports the diagnosis of SO but also helps distinguish SO from other uveitic entities, such as Vogt–Koyanagi–Harada disease, in which ICGA often shows a more diffuse pattern of hypofluorescence. In our patient, the identification of hypofluorescent spots on ICGA, in the context of prior vitrectomy and endophthalmitis, strongly favored SO over alternative diagnoses. OCT, especially enhanced depth imaging OCT (EDI-OCT), provides a quantitative biomarker of disease activity. In acute SO, OCT shows marked diffuse choroidal thickening, sometimes accompanied by an undulating appearance of the thickened choroid and blurring or loss of the normal choroidal vascular lumen-like structures (17). These changes reflect choroidal vascular engorgement, edema, and granulomatous infiltration. Our patient’s initial imaging findings were consistent with these characteristic ICGA and OCT features of SO. Following treatment, subfoveal choroidal thickness (SFCT) decreased, choroidal vascular structures became clearly discernible, and retinochoroidal edema diminished, all of which were consistent with the expected recovery course in SO. Thus, multimodal imaging facilitates early detection, accurate diagnosis, monitoring of treatment response, and prevention of recurrence in SO. OCT is particularly valuable as a non-invasive tool. SFCT measured by OCT correlates well with clinical disease activity in SO. After effective immunosuppressive or corticosteroid therapy, SFCT decreases significantly, paralleling improvements in visual acuity and anterior chamber inflammation. Notably, a sudden increase in choroidal thickness may precede clinical exacerbation by days to weeks, providing an early warning sign of relapse. This is particularly useful when ICGA is unavailable or angiography is contraindicated (e.g., allergy to fluorescein sodium).

Medical therapy for SO includes corticosteroid therapy and immunomodulatory therapy (IMT) (20, 21). Chronic systemic corticosteroid therapy at supraphysiologic doses, often required for treating SO, is associated with adverse effects in multiple organ systems, including worsening of diabetes mellitus, obesity, increased susceptibility to infections, aseptic necrosis of the hip, glaucoma, and cataract (22). Moreover, discontinuation of steroid therapy may potentially lead to rebound inflammation, even when tapered slowly (23), and steroid therapy alone may be insufficient for controlling chronic or recurrent SO (24). Immunomodulatory therapy has been shown to improve outcomes for patients with SO when used as an adjunct to corticosteroids (25). A large multicenter retrospective study of 130 patients reported that 70.7% of SO cases required IMT, suggesting that corticosteroids alone are inadequate for SO treatment (26). Furthermore, research indicates that longer cumulative durations of IMT and drug-free remission are correlated with lower rates of vision loss and better visual prognoses (27). Early aggressive treatment with immunomodulators may prevent photoreceptor mitochondrial oxidative stress, improve long-term visual outcomes, and minimize ocular complications (28). Adalimumab was approved by the U.S. Food and Drug Administration in 2016 for the treatment of non-infectious intermediate, posterior, and pan-uveitis (29, 30). Soheilian et al. (31) documented the use of adalimumab in a patient with refractory SO following phakic intraocular lens implantation. Hiyama et al. (32) demonstrated the efficacy of adalimumab in patients with steroid or immunosuppressant-refractory SO, particularly in those with glaucoma, for whom long-term steroid therapy should be avoided. The patient in the present case is relatively young and has a significant history of fungal endophthalmitis. In developing the treatment strategy, the foremost objective was to promote the recovery of visual function. However, it was equally critical to control potential complications associated with prolonged corticosteroid therapy, including reactivation of fungal infection, diabetes, and glaucoma. Although conventional steroid-sparing immunomodulatory agents such as methotrexate, azathioprine, or mycophenolate mofetil are commonly used in autoimmune uveitis, several considerations led us to choose adalimumab in this specific setting. First, conventional immunosuppressants typically require several weeks to months to achieve full efficacy, whereas adalimumab can induce a more rapid anti-inflammatory response—a crucial advantage given the active macular choroidal and optic disk edema that posed an imminent threat to vision. Second, the patient’s prior fungal endophthalmitis raised concern about broad and prolonged immunosuppression associated with conventional agents; adalimumab, as a targeted TNF-*α* blocker, allows more focused immunomodulation and has been used successfully in patients with a history of controlled infections under careful monitoring. Third, the patient was at risk of steroid-induced glaucoma and diabetes. In previous reports of refractory sympathetic ophthalmia, adalimumab has demonstrated an acceptable safety profile in young uveitis patients, particularly when long-term corticosteroid therapy should be avoided. Therefore, we opted for a regimen combining corticosteroids with adalimumab. The patient showed significant symptomatic improvement within 2 weeks of treatment. Subsequently, as treatment continued, gradual resolution of macular, choroidal, and optic disk edema was observed. Given the mild initial presentation and favorable therapeutic response, the medications were gradually tapered and discontinued after achieving disease stability. No recurrence was observed during a 1-year follow-up period.

## 4 Conclusion

SO is a sight-threatening condition that typically involves both eyes. Active intervention aimed at preserving the injured eye, protecting the fellow eye, and maintaining optimal visual function represents an important cornerstone of management. Immunomodulatory therapy has been shown to play a transformative role in the treatment of SO by enabling some patients to achieve remission without steroids or on low-dose steroids, potentially contributing to favorable visual outcomes in both eyes. This approach may require not only precise adjustment of immunomodulatory agent dosages to balance efficacy and side effects, but also the use of multimodal imaging techniques—including OCT and ICGA—to diagnose and monitor clinical inflammatory manifestations, thereby guiding treatment decisions. Furthermore, long-term follow-up appears equally essential to reduce the risk of recurrence. While these observations are derived from a single case and cannot be generalized, they suggest that a combination of corticosteroids and adalimumab may be a safe and effective option for selected patients with SO and a history of fungal endophthalmitis.

## Data Availability

The original contributions presented in the study are included in the article/supplementary material, further inquiries can be directed to the corresponding author.
